# Decreasing predictability of visual motion enhances feed-forward processing in visual cortex when stimuli are behaviorally relevant

**DOI:** 10.1007/s00429-016-1251-8

**Published:** 2016-06-22

**Authors:** Thilo Kellermann, Ruben Scholle, Frank Schneider, Ute Habel

**Affiliations:** 1 Department of Psychiatry, Psychotherapy and Psychosomatics, Uniklinik RWTH Aachen, RWTH Aachen University, Pauwelsstr. 30, 52074 Aachen, Germany; 2 JARA-BRAIN Institute 1: Structure Function Relationship, 52428 Jülich, Germany

**Keywords:** Effective connectivity, Dynamic causal modeling (DCM), Predictive coding, Hierarchical processing, Prediction error

## Abstract

Recent views of information processing in the (human) brain emphasize the hierarchical structure of the central nervous system, which is assumed to form the basis of a functional hierarchy. Hierarchical predictive processing refers to the notion that higher levels try to predict activity in lower areas, while lower levels transmit a prediction error up the hierarchy whenever the predictions fail. The present study aims at testing hypothetical modulatory effects of unpredictable visual motion on forward connectivities within the visual cortex. Functional magnetic resonance imaging was acquired from 35 healthy volunteers while viewing a moving ball under three different levels of predictability. In two different runs subjects were asked to attend to direction changes in the ball’s motion, where a button-press was required in one of these runs only. Dynamic causal modeling was applied to a network comprising V1, V5 and posterior parietal cortex in the right hemisphere. The winning model of a Bayesian model selection indicated an enhanced strength in the forward connection from V1 to V5 with decreasing predictability for the run requiring motor response. These results support the notion of hierarchical predictive processing in the sense of an augmented bottom-up transmission of prediction error with increasing uncertainty about motion direction. This finding may be of importance for promoting our understanding of trait characteristics in psychiatric disorders, as an increased forward propagation of prediction error is assumed to underlie schizophrenia and may be observable at early stages of the disease.

## Introduction

Current views about the general principle of the functioning of the brain emphasize the importance of predictions that are generated by the central nervous system. According to these views the hierarchical organization of the (human) brain plays a fundamental role in implementing this predictive mode of operation (Rao and Ballard 1999; Friston and Kiebel 2009; Friston 2010; Hohwy 2013; Clark 2013). Sensory information enters the system at low hierarchical levels while predictions of these sensory inputs are represented in higher levels. The architecture of the visual system of primates, for example, accommodates such a hierarchical structure in that ascending (or feed-forward) pathways predominantly originate in superficial layers of lower regions and terminate in layer IV of the hierarchically higher areas. Conversely, descending (or feedback) projections from higher to lower regions generally originate in deep pyramidal cells of layer V of the higher region while ending in layer I and VI of the lower area (Mumford 1992; Felleman and Van Essen 1991). Although recent tracer studies were able to show that the hierarchy proposed by Felleman and Van Essen is correct in most aspects, some restrictions have to be made. First, quantitative methods slightly rearranged the level of some visual areas within the hierarchical order, for example the position of the frontal eye fields (FEF). Second, the definition of feed-forward and feedback pathways originating mainly in the supra- and infragranular layers, respectively, is less strict. But it is nevertheless an appropriate indicator of the direction of connections (Barone et al. 2000; Vezoli et al. 2004; Markov et al. 2014).

Hierarchical predictive coding has been proposed as an explanation for extra-classical receptive-field effects in the visual cortex (Rao and Ballard 1999). This scheme assumes that redundancy in encoding sensory input is reduced by modeling its statistical regularities. Instead of propagating all inputs from one level to the next, only residuals or errors containing the deviation of the input from the prediction are passed up the hierarchy. Predictions provided by higher regions are used to explain the input in lower regions via their backward projections. The same concept underlies the “Bayesian brain hypothesis” (Knill and Pouget 2004) which emphasizes the probabilistic nature of such iterative processes of sensing and predicting. Friston (2010) has proposed a unified theory—the free-energy principle—which states that self-organizing systems need to minimize their free-energy to survive (Friston and Stephan 2007). In the current context it is important to know that under certain simplifying assumptions, minimizing free-energy is equivalent to minimizing prediction error with both leading to “Bayes optimal” results. In this way it is assumed that generative models, which try to infer the underlying causes in the (outside) world from sensory inputs, are implemented, tested and updated in the brain by hierarchical predictive processing (Clark 2013).

In a previous study we investigated the effective connectivity of the cerebellum with visual areas during an attention-to-motion task (Kellermann et al. 2012). The pattern of modulatory inputs of attention to the uniform and therefore, highly predictable motion fitted well with both the presumed role of the cerebellum as a state estimator (also) in perception (Paulin 2005; O’Reilly et al. 2008) and the notion of hierarchical predictive processing. The posterior parietal cortex (PPC) sent its outputs via crus I of the cerebellum to the lower region V5, where the latter connection, namely from crus I to V5, was enhanced during attention to the predictable stimuli. Conversely, we found a suppression of the feed-forward connection from V5 to PPC at the same time, i.e., during attention to predictable motion. The present study aimed at testing specific hypotheses derived from hierarchical predictive processing during unpredictable visual motion by means of dynamic causal modeling (DCM) for functional magnetic resonance imaging (fMRI). Compared to our previous investigation, in which top-down (or goal-directed) attention was manipulated, the present study presumes attentional effects as a result of stimulus-driven feed-forward effects, with randomly behaving stimuli capturing more attention than predictable ones. In contrast to predictable stimuli, unpredictable visual motion would be associated with an enhanced strength of feed-forward connections, e.g., from V1 to V5 or from V5 to PPC. Goal-directed attention has been associated with the optimization of expected precision in the sense of an enhanced modulatory effect of attention on the self-connections of higher order nodes (Feldman and Friston 2010; Kok et al. 2012). This modulatory effect on the self-connections was also tested at the level of V5 in the present study with the distinction that attention was rather stimulus-driven as compared to goal-directed. While this enhancement of bottom-up processing reflects the message passing of prediction error up the hierarchy, a simultaneous down regulation of top-down influences might be conceivable. This effect might reflect reduced top-down “explanations” of sensory inputs in lower regions (e.g., V1) by representations in higher areas (e.g., V5).

The main hypothesis pursued in this study states that a Bayesian model selection procedure among a large space of dynamic causal models would yield highest probability for a model (or a family of models) in which stimulus unpredictability positively modulates forward connectivity and/or negatively modulates backward connectivity within the visual hierarchy. The nodes whose hierarchical connections we chose to examine were primary visual cortex V1, motion-sensitive visual cortex V5 and posterior parietal cortex (PPC).

## Experimental methods

### Subjects

The complete sample comprised 37 healthy, right-handed subjects, two of whom were excluded due to excessive head motion (translation of more than 3 mm). The remaining 35 participants (21 males, 14 females) had no history of neurological or psychiatric illness and were aged between 18 and 41 years (mean 27.2 years, SD 4.7 years). All subjects gave written informed consent prior to participation in the study. The study adhered to the standards provided by the Declaration of Helsinki regarding ethical principles for medical research involving human subjects and the local Institutional Review Board approved the protocol.

### Stimuli and task

Visual stimuli were presented by means of an MR-compatible goggles system (Resonance Technology Company Inc., Los Angeles, USA). The visible screen covered approximately 25° × 19° of the visual field of the subjects with a resolution of 800 × 600 pixels. Controlling and timing of stimuli was achieved using the Presentation software (Neurobehavioral Systems Inc., Berkeley, USA). The visual stimuli consisted of a white frame (~24° × 16°) on a black background containing a white filled circle (~2° in diameter). During the baseline condition the white circle (or “ball”) was presented stationary within the white frame where the starting point at the beginning of each run was the center of the screen. In each of the 30 experimental blocks the ball moved with a constant speed of ~6° per second for 20, 20.5 or 21.5 s without leaving the frame. Between two subsequent experimental blocks a baseline with a mean length of 10 s was inserted in which the ball stopped moving and stayed at the last position of the preceding block. The following experimental block started with a jitter of 0, 0.5 or 1.5 s and the ball began moving again starting from its last position.

**Fig. 1:**
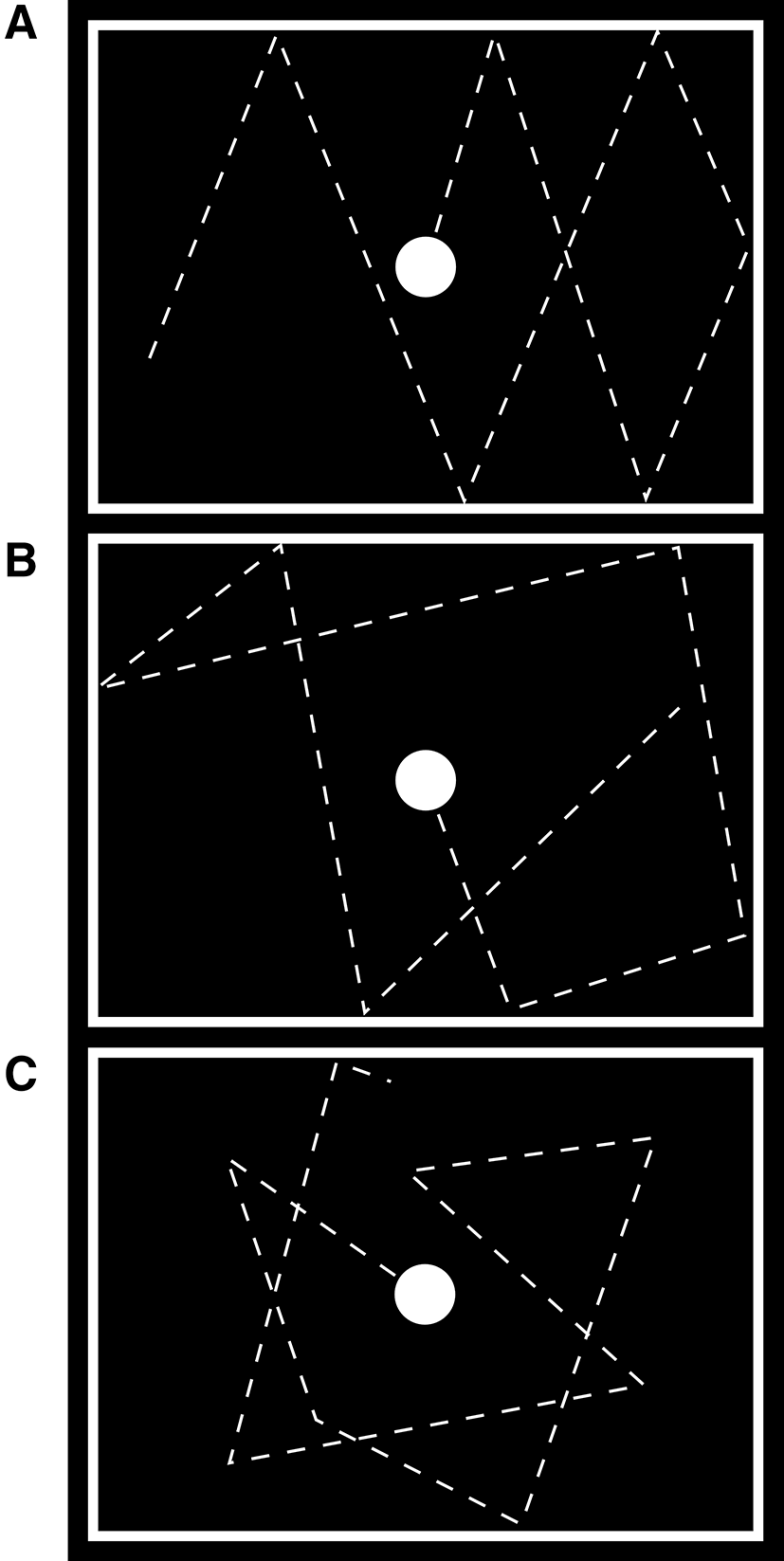
Illustration of the exemplary trajectories of the visual motion. Each panel depicts the movement of the *white circle* roughly during the first 10 seconds of a block. The different panels show exemplary trajectories of the *ball* during the predictable** a**, random **b** and arbitrary **c** condition

The 30 experimental blocks of one run were divided into three different conditions, where the sequential order was pseudo-randomized and the durations as well as the jitters were counterbalanced. During the PREDICTABLE condition the ball changed its direction of motion if and only if it touched the border of the frame where the angle of dip corresponded to the emergent angle. Thus, the trajectory of the ball was predictable because of its resemblance of a ball bouncing from a cushion of a pool table. The RANDOM blocks were less predictable than the aforementioned condition since the emergent angle—when the ball rebounded from the cushion—varied randomly and thus did not correspond to the incident angle with the constraint that the ball stayed within the frame. Finally, the ARBITRARY condition was the least predictable because changes in direction of motion not only occurred with contacts of the ball with the cushion but also in random intervals in the middle of the frame (see Fig. 1). Hence, the predictability of the motion decreased from the PREDICTABLE over the RANDOM to the ARBITRARY condition. It should be noted, however, that the ARBITRARY condition differed from the other two conditions also in terms of the number of motion direction changes which occurred about 1.6 times more often than in one of the other two conditions. On average, one session contained 184.8 (SD 3.6) changes in the PREDICTABLE condition, 183.7 (SD 9.2) changes in the RANDOM condition and 288.9 (SD 12.2) changes in the ARBITRARY condition. This confounding effect and its impact on the interpretability of the results will be considered in the discussion of the data.

In each of the two runs per participant the subject was instructed to keep track of the ball and to attend to its changes in direction of motion. In other words subjects were requested to pursue the moving ball overtly with their eyes and to look out for motion direction changes. The two runs differed from each other only in the response-mode where the participant had to indicate each (perceived) change in motion direction by a button-press with the right index finger in the “active” run, whereas the subject just had to keep track of the ball and attend to motion direction changes (without any motor response) in the “passive” run. To familiarize the subjects with the stimuli, the passive run preceded the active one for most of the participants (21 of the 35; 13 males, 8 females). To exclude, however, the possibility that any (main or interaction) effects of the response-mode (active vs. passive) might be due to their mere sequential order, the remaining 14 subjects (8 males, 6 females) were measured with the reversed order.

### Data acquisition

Functional magnetic resonance imaging (fMRI) was performed using a Siemens Trio 3T MRI scanner. In each of the two runs per subject 515 functional images were acquired using a T2*-weighted echo-planar imaging (EPI) sequence covering the whole brain with 33 axial slices having a thickness of 3.4 mm (gap between slices 0.51 mm). Each slice had a resolution of 64 × 64 pixels and a field of view of 200 × 200 mm^2^, resulting in a voxel size of 3.125 × 3.125 × 3.4 mm^3^. The echo-time (TE) was 30 ms, the flip-angle amounted to 75° and the repetition time (TR) was 1800 ms, which resulted in an acquisition time of 15 min and 45 s per functional run. The first three images of each run were discarded due to T1 stabilization effects. After the two functional runs an anatomical image was acquired with a T1-weighted magnetization prepared rapid gradient echo (MPRAGE) sequence yielding a resolution of 1 × 1 × 1 mm^3^ (TR: 1900 ms, TE: 2.52 ms, flip-angle: 9°).

### Data preprocessing and general linear model analyses

Preprocessing and analyses of fMRI data were performed in SPM8 (Wellcome Trust Centre for Neuroimaging, London) implemented in Matlab 8 (The MathWorks). The remaining 512 functional images of each run were realigned using the two-pass procedure implemented in SPM. Anatomical scans were aligned to the resulting mean EPI of each run and normalization parameters were obtained using the unified segmentation approach (Ashburner and Friston 2005). The functional time-series were transformed into the standard space defined by the Montreal Institute of Neurology (MNI) by applying the normalization parameters to the time-series. Normalized images were resampled at a resolution of 2 × 2 × 2 mm^3^ and spatially smoothed with an isotropic Gaussian kernel of 8 mm full width at half-maximum.

The two runs per subject were modeled by convolving the boxcar functions of the three conditions per run with the canonical hemodynamic response function. The above mentioned baseline during which the ball was presented stationary served as implicit (i.e., not explicitly modeled) low-level baseline (see “Stimuli and task”). The resulting six (2 runs by 3 conditions) predictors were used as regressors in a general linear model (GLM), where the realignment parameters and intercepts of each run served as covariates of no interest. Low-frequency drifts were removed by a high-pass filter with a cut-off period of 128 s and temporal autocorrelations were accounted for by removing the estimated first-order autoregressive effects of the time-series. The resulting six volumes of interest with the parameter estimates per participant were subjected to a 3 × 2 mixed-effects ANOVA at the group level with predictability (PREDICTABLE, RANDOM and ARBITRARY) and response-mode (ACTIVE and PASSIVE) as fixed effects factors. Variance components were specified to account for heteroscedasticity (between conditions and subjects, where the latter was implemented as random-effects factor) and dependencies among within-subject observations. The threshold for rejecting the null-hypothesis was set to *p* < 0.001, family-wise error corrected at the voxel level for multiple comparisons per contrast with an additional extent threshold of 100 continuous voxels.

### Dynamic causal modeling

Dynamic causal modeling (DCM) was performed using DCM10 as implemented in SPM8. In short, with DCM one models observed data from coupled brain regions in terms of their endogenous connectivity structure, driving inputs of experimental conditions and modulatory inputs of these conditions on the connectivities between nodes. The observed fMRI data is modeled by an explicit forward model specifying how the measured signal was caused at the neuronal level (Friston et al. 2003). Most importantly, the same data is then explained by a set of different competing models all of which are based on the same forward model but differ with respect to their connectivity structure. The interaction of the exogenous inputs (direct or modulatory) to the system and the neuronal states is modeled by means of a bilinear differential equation as shown in the formula below. The variable *x* represents the neuronal states in the *n* nodes (or regions). The *n* × *n* matrix *A* contains the time-invariant coupling parameters for the connections between nodes (if the respective connection is present) as well as the self-connections. The three-dimensional *n* × *n* × *m* matrix *B* entails the parameters of the modulatory inputs of the *m* experimental inputs (denoted by *u*) on the connections between nodes as well as on the self-connections. Finally, matrix *C* is of size *n* × *m* and comprises the direct input parameters of the *m* experimental conditions on the *n* nodes.

1 $${\raise0.7ex\hbox{${{\text{d}}x}$} \!\mathord{\left/ {\vphantom {{{\text{d}}x} {{\text{d}}t}}}\right.\kern-0pt} \!\lower0.7ex\hbox{${{\text{d}}t}$}} = \left( {A + \sum\limits_{i = 1}^{m} {u_{i} B^{(i)} } } \right)x + {\text{Cu}}$$

Different competing models can be specified by inclusion (1) or omission (0) of one or more of the parameters in the matrices *A*, *B* and *C*, resulting in an exhaustive model space comprising 2^(*n* × *n*) × (*m* + 1) + (m × *n*)^ models in the case when all combinations of connections and inputs shall be modeled. Usually only a substantially smaller subset of “plausible” models is considered in the model space to keep inversion of all models in the space computationally feasible. Inference made during Bayesian model selection (BMS), however, refers only to the tested models within the space and does not extend to any model of the exhaustive space that is not included. The competing models can then be compared to each other based on their log-evidence approximated with their variational free-energy, from which a posterior probability for each model can be derived reflecting the relative evidence of that model given the data.

Time-series were extracted for analyses of effective connectivity from primary visual cortex (V1), motion-sensitive extra-striate cortex (V5) and posterior parietal cortex (PPC). Coordinates of the regions were based on the group analysis of a contrast comparing all moving stimuli against the low-level baseline (not reported). Individual coordinates were then found by jumping to the nearest local maximum in the respective first-level contrast. The first eigenvariate of all suprathreshold voxels (*p* < 0.01 uncorrected) within a sphere of 5 mm radius was used to represent the time-series of the respective region. High-pass filtering was applied to these data as specified above and the variance explained by the realignment parameters and the session intercepts was removed. The direct inputs used in dynamic causal modeling (DCM) were slightly modified compared to the GLM in the sense that the first predictor included all moving visual stimuli (i.e., PREDICTABLE, RANDOM and ARBITRARY, henceforth MOTION), the second one contained the two non-predictable conditions (RANDOM and ARBITRARY, henceforth UNPREDICTABLE) and the last was identical to the ARBITRARY regressor. Thus, the projection space was identical to the GLM analysis, where this modeling more directly reflects the additional effects of decreasing predictability.

**Fig. 2:**
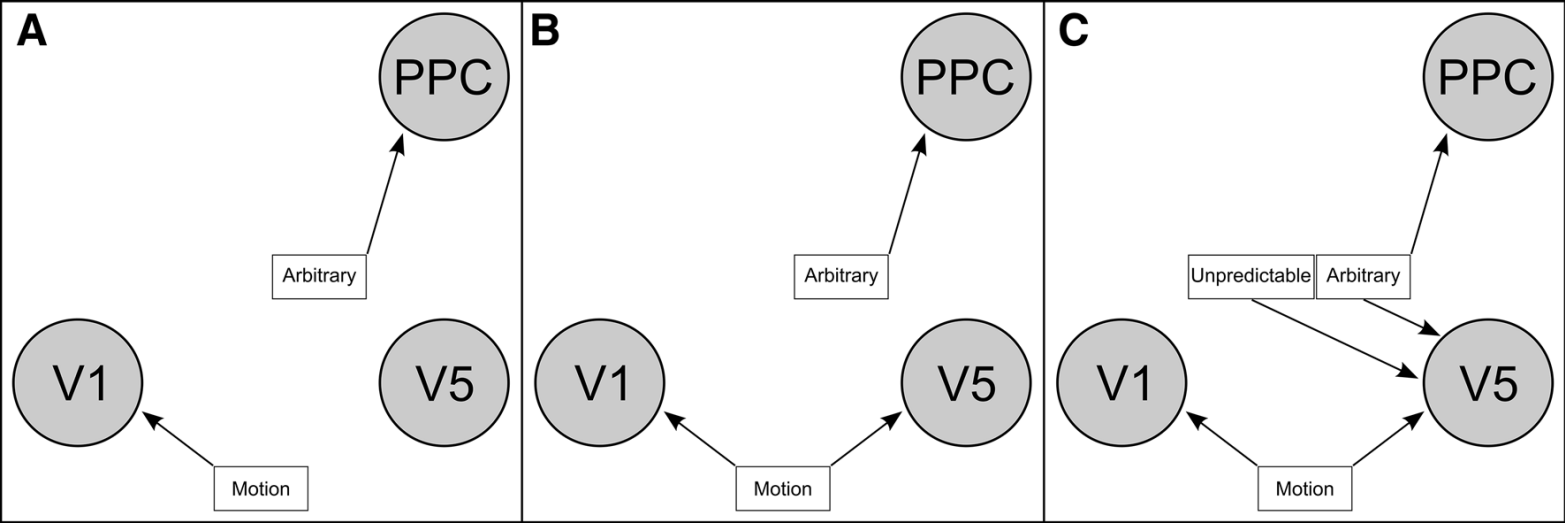
Different input structures of the three model families. Overview of the different driving input structures of the three model families examined in the Bayesian model selection. Each of the three families contained 256 models with different combinations of the modulatory inputs of unpredictable and/or *arbitrary* stimuli on the connections between *the nodes*

Bayesian model selection (BMS) was performed among a set of models to test the hypothesis that increasing unpredictability of visual motion positively modulates feed-forward connections. This main BMS was preceded by pre-selection of models which is described in detail with respect to its rationale, procedure and results in the paragraphs below. For the main BMS the endogenous connectivity structure between the three nodes consisted of reciprocal connections between V1 and V5 on the one hand and between V5 and PPC on the other, i.e., two feed-forward (V1 → V5 and V5 → PPC) and two backward (V5 → V1 and PPC → V5) connections. One family of models within the model space had a driving input of MOTION on V1 and another direct input of ARBITRARY stimuli on PPC (Fig. 2a). One other family had an additional direct input of MOTION on V5 (Rodman et al. 1989; Girard et al. 1992; Sincich et al. 2004) (see Fig. 2b). Based on previous model selection procedures concerning the effects of direct inputs on the three nodes (see below) we also included a family of models with the additional direct inputs of UNPREDICTABLE on V5 and of ARBITRARY on V5 and PPC (see Fig. 2c). Each family comprised 256 models reflecting the 2^(2×4)^ possible modulatory effects of the two conditions UNPREDICTABLE and ARBITRARY on the four connections described above. Common to each single model was the modulatory effect of MOTION on the V1 → V5 connection. Thus far, the model space consisted of 768 models per subject and session. However, we also tested a change in the synaptic gain of V5 due to either UNPREDICTABLE or ARBITRARY stimuli, which tripled the number of models in the model space to 2304. This is the model space which is referred to in the results section.

In what follows we describe a two-step pre-selection procedure that was performed prior to the main BMS described above. Because the main BMS depended on the results of this pre-selection, results of this procedure are already included here, whereas the results of the main BMS can be found in the results section. The first pre-selection of dynamic causal models (DCMs) served the identification of direct inputs of the three conditions (MOTION, UNPREDICTABLE and ARBITRARY) on one or more of the three nodes. The rationale for this procedure was the negligence of other regions that may exert particularly top-down effects on the modeled system, which may be associated with enhanced salience or saccadic eye movements during the ARBITRARY condition. If such effects of non-included regions (e.g., the frontal eye fields or superior colliculi) exist, one way to model these in a reduced system would be as direct inputs to one or more of the included nodes. The sequential testing of subspaces of models was necessary to keep the computational burden for the main research question feasible. Although sequential testing cannot equivalently replace a test of all combinations of parameters, we pursued this suboptimal strategy to test several different direct inputs while keeping the computational load manageable at the same time. Sequential testing of several subspaces is rather unproblematic for fixed-effects (FFX) Bayesian model selection (BMS) as long as all models in Occam’s window are considered in each selection. Random-effects (RFX) BMS, however, may yield inconclusive results when used sequentially (Penny et al. 2010). It should be emphasized that sequential model selection still bears the risk that there are models with a combination of parameters not tested during one of the BMS which are superior to the winning models of the restricted spaces tested in this study. In other words, the main BMS only tests for those models that are included in that selection and it does not make any inference on models outside that space. Therefore, the pre-selection can only be regarded as some sparse evidence for the direct inputs.

In a first consideration we concentrated on the combinations how UNPREDICTABLE and/or ARBITRARY might perturb the system at V5 and/or at PPC, reducing the number of possibilities to 2^(2×2)^ = 16. Because the visual input to the system did not change with respect to any other property than motion (even the low-level baseline included a static view of the visual stimuli), we also tested if MOTION exerted a direct influence on V1. Theoretically, the input to V1 might have been a constant across the entire time-series, where the effect of MOTION, for example, is realized as an exclusively modulatory input (e.g., on the connection from V1 to V5). Therefore, complete model space of this first pre-selection procedure included 2^(2 × 2)+1^ = 32 models reflecting all possible combinations of direct inputs of MOTION on V1 and/or UNPREDICTABLE and/or ARBITRARY on V5 and/or PPC (see Table 1A where these 5 direct inputs are indicated with an X).

**Table 1:** A denotes the direct inputs that were switched on and off with an ‘X’ for a first pre-selection. This resulted in an input structure denoted with a ‘1’ in B. Then another selection was performed using this input structure while switching those inputs denoted with an ‘X’ in B. C shows the winning input structure of this pre-selection

	MOTION	UNPREDICTABLE	ARBITRARY
A			
V1	X	0	0
V5	0	X	X
PPC	0	X	X
B			
V1	1	0	0
V5	X	1	1
PPC	X	0	1
C			
V1	1	0	0
V5	1	1	1
PPC	0	0	1

The endogenous connectivity structure was the same as for all models, namely reciprocal connections between V1 and V5 and reciprocal connections between V5 and PPC. In addition, the models shared the modulatory input of MOTION on the V1 → V5 connection. With respect to the modulatory inputs of UNPREDICTABLE and/or ARBITRARY on any of the endogenous connectivities, we included all parameters assuming that their inclusion rendered the need for additional parameters reflecting direct inputs rather improbable. BMS among these 32 models using fixed effects for inference indicated strong evidence in favor of the model with four simultaneous driving inputs, namely MOTION → V1, UNPREDICTABLE → V5, ARBITRARY → V5 and ARBITRARY → PPC with a posterior probability exceeding 99.99 %.

In a second step during pre-selection, we asked for the plausibility of a direct input of MOTION on V5 and/or PPC, keeping other direct inputs, endogenous connectivity and modulatory inputs from the winning model in the first step. Therefore, we tested the winning model of the BMS above against the three other models that allowed MOTION to drive either V5 or PPC or both V5 and PPC (Table 1B). The winning model of this BMS (again using fixed-effects) clearly outperformed the competing three models with a posterior probability exceeding 99.99 % and indicated that MOTION had a driving input in V1 and V5, UNPREDICTABLE had a direct input in V5 and ARBITRARY had a direct input in V5 and PPC (see Table 1C; Fig. 2c). The result of this pre-selection was the reason for inclusion of a whole model family in the main BMS with this rather complicated input structure which is depicted in Fig. 2c.

## Results

### Descriptive results of the behavioral data

During the response-mode session subjects pressed the button on average 183.4 times (SD 7.5) in the PREDICTABLE condition. In the RANDOM condition subjects gave on average 173.7 responses (SD 12.0), whereas the average number of button presses in the ARBITRARY condition yielded 235.2 events (SD 24.5). Due to the frequent number of motion direction changes particularly in the ARBITRARY condition (but also occasionally in the RANDOM condition when the ball was located near one of the edges) a distinct accuracy assignment was not possible.

### Two-way ANOVA predictability × response-mode

**Fig. 3:**
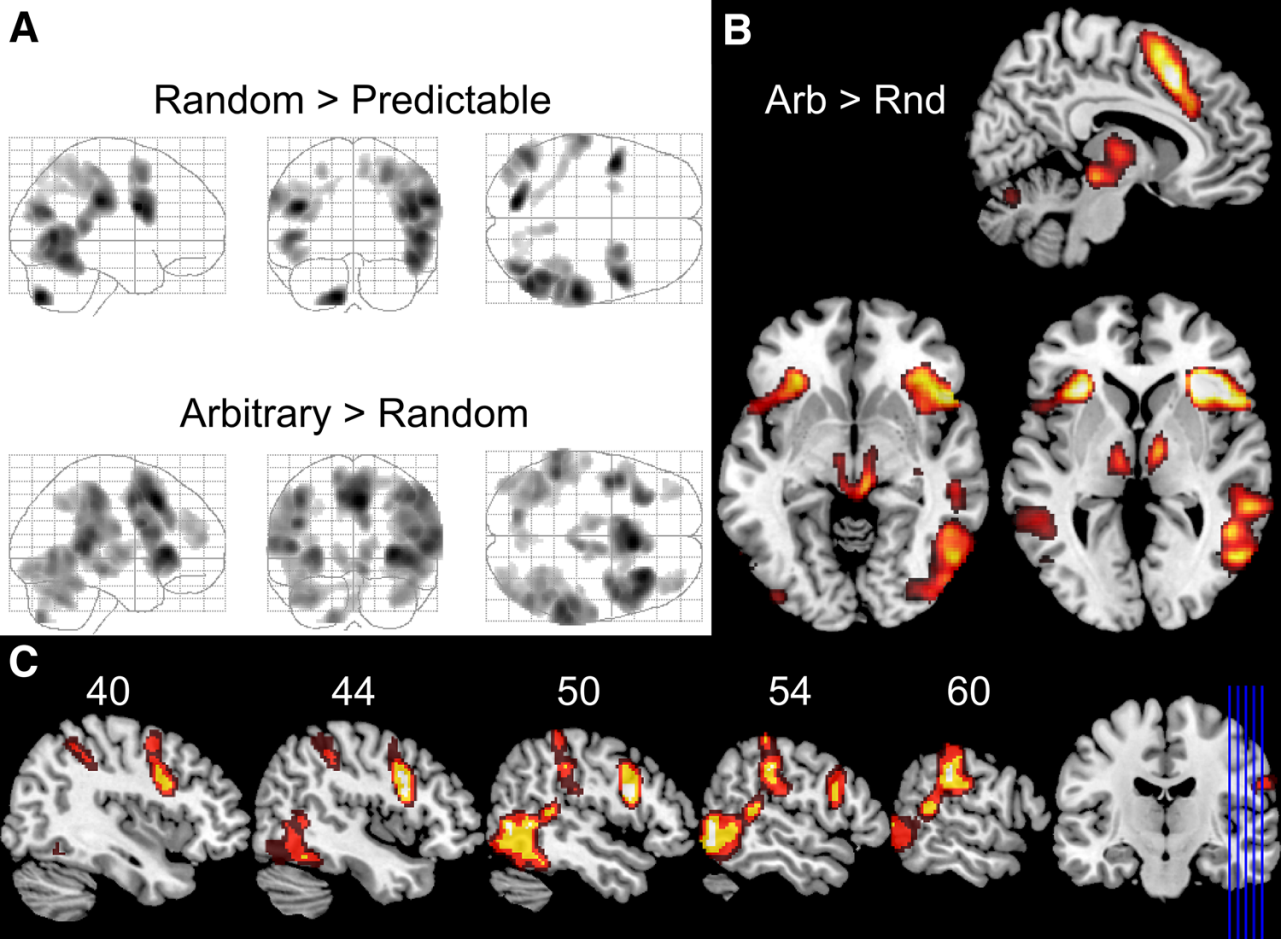
Depiction of activation differences between the levels of predictability.** a** Maximum intensity plots of the contrast Random > Predictable motion (*upper panel*) and *Arbitrary > Random motion* (*lower panel*).** b** Selected sections of the *Arbitrary (Arb) > Random (Rnd)* contrast showing clusters inter alia in the dorsomedial prefrontal cortex, the thalamus and brain stem. The sagittal section in the *upper right panel* shows significant activations at the level of *x* = 8 mm, whereas the axial slices at the bottom show those at the level of *z* = −6 mm (*left*) and *z* = 2 mm (*right*).** c** Sagittal slices from the right hemisphere showing the conjunction of the two contrasts depicted in** a**. Numerals above the slices indicate the distance in millimeters (*z*-coordinates) from the midline. The *blue lines* on the coronal slice on the right illustrate the levels of the sagittal slices. All images were thresholded at *p* < 0.001 corrected at the voxel level and an extent threshold of 100 contiguous voxels

Activation of the dorsal visual stream of all moving visual stimuli against baseline (MOTION contrast) covered the whole dorsal visual stream as well as the supposed human homologue to the frontal eye fields (FEF) and a large part of the cerebellum (results not shown). The main effect of predictability is confined to the one-tailed *t* contrasts RANDOM > PREDICTABLE and ARBITRARY > RANDOM and their conjunction (see Fig. 3) which was performed as test against the conjunction null-hypothesis (Nichols et al. 2005).

The former of the two comparisons yielded a slightly right lateralized network as indicated by a negative lateralization index of −0.39. This index was assessed by subtracting the number of suprathreshold voxels in the right hemisphere from those in the left hemisphere and dividing this difference by the total number of suprathreshold voxels. This network comprised bilateral extra-striate cortices (V5 and middle occipital gyrus), bilateral frontal eye fields (FEF), right inferior frontal gyrus and left inferior precentral gyrus. The large cluster in the right hemisphere comprising V5 extended dorsally to superior temporal gyrus (STG) and the supramarginal gyrus, thus also covering the temporo-parietal junction (TPJ). The homologue areas in the left hemisphere of the last cluster corresponded to isolated activations in V5 and supramarginal gyrus. In addition, lobule VIIa of the left cerebellar hemisphere was more active during the RANDOM as compared to the PREDICTABLE condition (see Table 2).

**Table 2:** Activation clusters for the comparison random versus predictable motion

Anatomical label	Anatomy toolbox	Cluster size	*t* score	MNI coordinates		
				*x*	*y*	*z*
Right supramarginal gyrus	IPC (PF)	5612	10.37	60	−28	32
Right middle temporal gyrus	hOC5 (V5)		9.94	54	−64	2
Right inferior temporal gyrus			9.67	48	−54	−14
Right middle temporal gyrus	IPC (PGp)		9.17	42	−72	22
Right inferior temporal gyrus			9.08	54	−68	−10
Right middle occipital gyrus	IPC (PGp)		9.07	40	−76	24
Right middle temporal gyrus			8.96	48	−54	4
Right middle temporal gyrus			8.76	56	−56	−2
Right superior temporal gyrus			8.50	58	−40	12
Right inferior parietal lobule			7.70	56	−32	56
Right supramarginal gyrus	Area 2		7.41	40	−36	44
Right inferior frontal gyrus (p. Opercularis)	Area 44	1678	10.12	48	8	24
Right middle frontal gyrus			8.38	28	6	54
Right middle frontal gyrus	Area 6		7.43	38	0	52
Right precentral gyrus			7.33	34	−2	50
Left supramarginal gyrus	IPC (PF)	904	8.45	−64	−22	36
Left inferior parietal lobule			6.69	−44	−38	42
			6.49	−20	−56	42
	hIP1		6.45	−36	−40	38
			6.13	−26	−48	38
Left precuneus	SPL (7A)		5.97	−12	−62	48
Left cuneus			5.78	−18	−72	36
Left middle temporal gyrus	hOC5 (V5)	865	9.09	−50	−68	2
Left inferior occipital gyrus	hOC5 (V5)		7.62	−46	−74	−12
Left precentral gyrus		464	10.49	−44	2	26
Left cerebellum	Lobule VIIb	347	10.63	−12	−76	−46
Left middle occipital gyrus	IPC (PGp)	263	7.48	−38	−82	20
Left middle frontal gyrus		105	7.09	−26	2	54

The comparison of ARBITRARY to RANDOM stimuli exhibited both similarities as well as differences to the aforementioned contrast. In general, the activation pattern was a bit more symmetrical (lateralization index −0.26), the common activated areas were spatially larger and other regions were recruited in addition, particularly dorsomedial prefrontal cortex (dmPFC) and subcortical nuclei in the thalamus and brain stem (a complete list of activated clusters is summarized in Table 3). The above mentioned large right hemispherical activation containing V5, STG and the supramarginal gyrus survived the statistical threshold again, where the cluster extended to more inferior brain areas comprising the fusiform gyrus (FFG) and even bestriding a local maximum within crus I of the right cerebellar lobule VIIa. The corresponding activation in the left hemisphere was fragmented into smaller clusters, but—apart from V5—also included crus I, FFG, STG and the supramarginal gyrus. A huge activation cluster comprised of more than 7000 voxels was likely the result of a merging of several smaller clusters as indicated by several local maxima (see Table 3). This large cluster in the (right) prefrontal cortex stretched inferiorly from the anterior insula over the inferior frontal and precentral gyri to the superior frontal gyrus, bilateral supplementary motor area (SMA) including dmPFC and the dorsal anterior cingulate cortex (dACC), sometimes referred to as mid-cingulate cortex (see Fig. 3b). The homologue areas in the left prefrontal cortex were constrained to the lateral parts but also comprised the anterior insula and inferior frontal and precentral gyri. Two more clusters were found more anterior at the cortical level, namely in bilateral middle frontal gyrus. At the subcortical level there was one supra-threshold cluster covering most of the bilateral thalamus as well as part of the tectum, particularly the colliculi superior. The test against the conjunction null-hypothesis of the two contrasts RANDOM > PREDICTABLE and ARBITRARY > RANDOM (see Fig. 3c) yielded the highest absolute lateralization index of −0.61 indicating a lateralization to the right hemisphere.

**Table 3:** Activation clusters of the comparison arbitrary versus random motion

Anatomical label	Anatomy toolbox	Cluster size	*t* score	MNI coordinates		
				*x*	*y*	*z*
Right middle temporal gyrus		7149	10.78	58	−40	10
Right superior temporal gyrus	IPC (PF)		10.35	66	−34	18
Right supramarginal gyrus	IPC (PFt)		9.84	54	−32	44
Right supramarginal gyrus	IPC (PF)		9.73	56	−32	50
Right middle temporal gyrus	hOC5 (V5)		9.07	52	−66	2
Right middle temporal gyrus			8.95	50	−52	0
Right inferior temporal gyrus			8.92	52	−66	−8
Right supramarginal gyrus	IPC (PF)		8.72	62	−28	32
Right fusiform gyrus			8.54	46	−50	−20
Right supramarginal gyrus	IPC (PFop)		8.50	60	−16	24
Right cerebellum	Lobule VIIa Crus I		8.19	38	−56	−30
Right SMA	Area 6	6332	12.18	4	16	48
Right insula lobe			11.79	32	26	4
Right insula lobe	Area 45		10.91	44	22	0
Right precentral gyrus			10.15	46	4	50
Right inferior frontal gyrus (p. Opercularis)			9.90	48	16	20
Right inferior frontal gyrus (p. Opercularis)	Area 44		9.90	50	12	20
Right precentral gyrus	Area 6		9.48	38	−2	50
Right inferior frontal gyrus (p. Opercularis)			8.92	42	10	32
Left SMA	Area 6		8.88	−12	6	64
Right superior frontal gyrus			6.94	24	12	60
Left middle cingulate cortex			6.04	−10	24	34
Left middle temporal gyrus		2717	10.20	−50	−44	10
Left superior temporal gyrus			9.47	−62	−44	12
Left inferior parietal lobule	hIP2		8.51	−50	−40	42
Left SUPRAMARGINAL Gyrus	IPC (PFcm)		8.28	−54	−40	24
Left supramarginal gyrus	IPC (PFcm)		8.27	−52	−44	30
Left supramarginal gyrus	IPC (PF)		8.24	−58	−38	24
Left supramarginal gyrus	IPC (PF)		8.12	−52	−38	34
Left superior temporal gyrus	IPC (PFcm)		7.51	−46	−32	20
Left supramarginal gyrus	IPC (PFop)		7.05	−58	−22	24
Left inferior parietal lobule	hIP1		6.24	−32	−46	38
Left inferior parietal lobule			6.14	−28	−52	44
Left cerebellum	Lobule VIIb (Hem)	2289	8.95	−16	−76	−46
Left cerebellum	Lobule VIIa Crus I		8.83	−32	−60	−32
Left cerebellum	Lobule VIIa Crus I		8.57	−40	−58	−32
Left cerebellum	Lobule VIIa Crus I		8.04	−34	−68	−26
Left cerebellum	Lobule VIIa Crus I		7.71	−44	−62	−24
Left inferior occipital gyrus	hOC4v (V4)		7.59	−42	−78	−14
Left inferior occipital gyrus	hOC4v (V4)		7.55	−40	−86	−12
Cerebellar vermis	Lobule VIIIa (Vermis)		7.02	0	−70	−34
Cerebellar vermis	Lobule VI (Hem)		6.66	6	−74	−22
Left cerebellum	Lobule VI (Hem)		6.56	−6	−78	−22
Cerebellar vermis	Lobule VIIIb		5.93	0	−60	−38
Left precentral gyrus	Area 6	2044	10.40	−42	−2	46
Left inferior frontal gyrus (p. Opercularis)	Area 44		10.33	−44	6	28
Left insula lobe			10.17	−32	22	2
Left insula lobe			10.15	−30	24	4
Left temporal pole			7.15	−50	14	−4
		1188	8.92	8	−26	−10
Right thalamus	Th-prefrontal		8.51	10	−12	4
Left thalamus	Th-prefrontal		7.97	−10	−14	0
Left thalamus	Th-prefrontal		7.88	−8	−16	−2
Left thalamus	Th-prefrontal		7.54	−12	−12	4
Right thalamus	Th-parietal		7.12	16	−18	12
			7.07	−4	−30	−6
			6.88	−6	−26	−8
			6.29	16	2	12
Right middle frontal gyrus		516	7.82	40	54	16
Right middle frontal gyrus			7.34	40	44	24
Right middle frontal gyrus			7.00	38	44	34
Left middle frontal gyrus		192	6.93	−34	50	12
Left middle frontal gyrus			6.29	−42	44	20

**Fig. 4:**
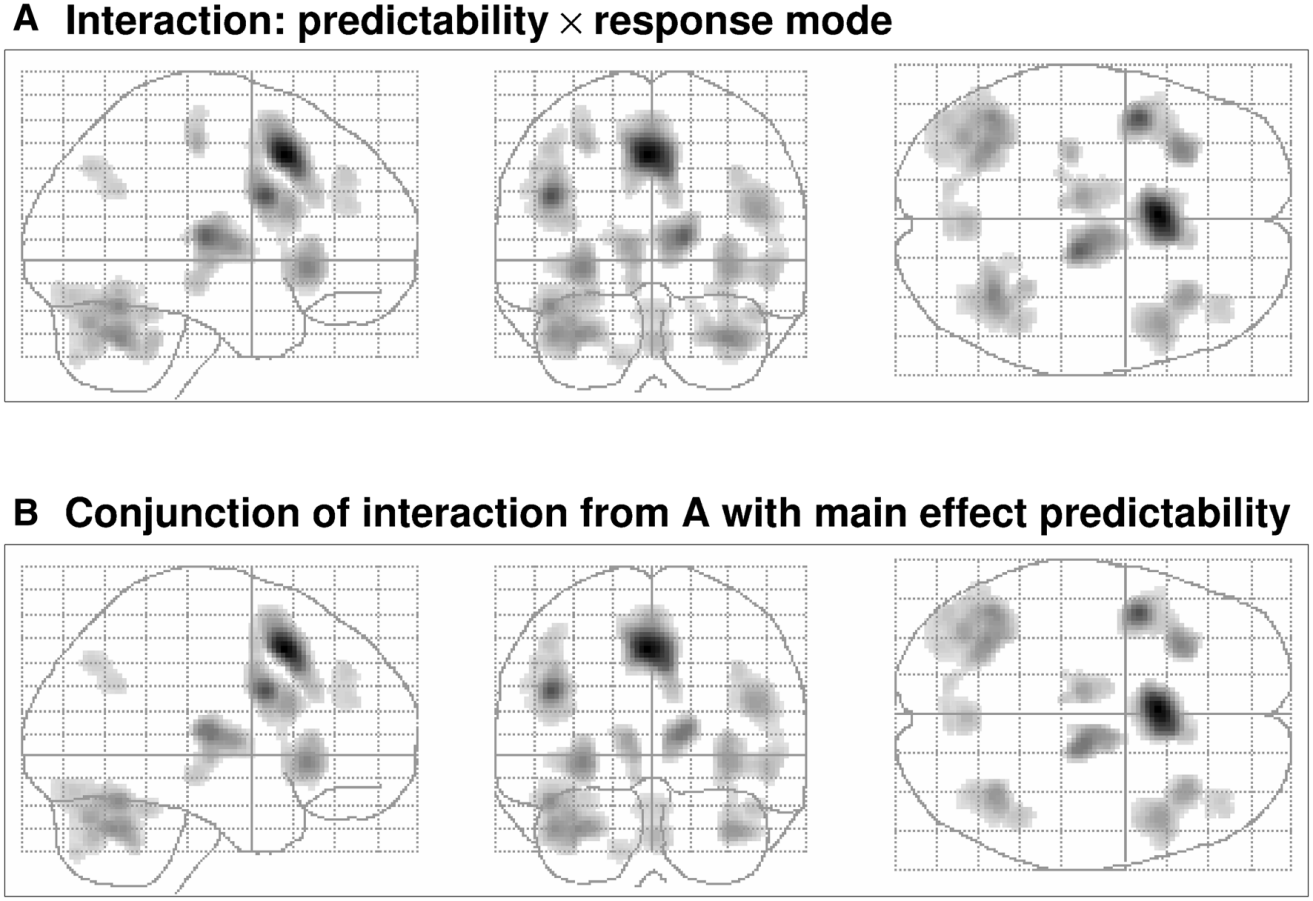
Maximum intensity pots of the predictable × response-mode interaction and its conjunction with the main effect predictability. Illustration of the similarity of the maximum intensity plots (MIP) of the predictable × response-mode interaction (**a**) and the conjunction of the same interaction with the main effect of predictability (**b**). Both MIPs were thresholded at *p* < 0.001 corrected at the voxel level and an extent threshold of 100 contiguous voxels

With respect to the predictability × response-mode interaction, it must be noted that 82.5 % of the suprathreshold voxels of this interaction are a subset of the main effect predictability (see Fig. 4). In other words, nearly all regions exhibiting an interaction effect differed also profoundly with respect to their responses to the predictability of visual motion, where less predictability was associated with more activity. More precisely, this interaction occurred in bilateral dmPFC (close to pre-SMA), bilateral thalamus and bilateral inferior frontal gyrus as well as insula.

**Fig. 5:**
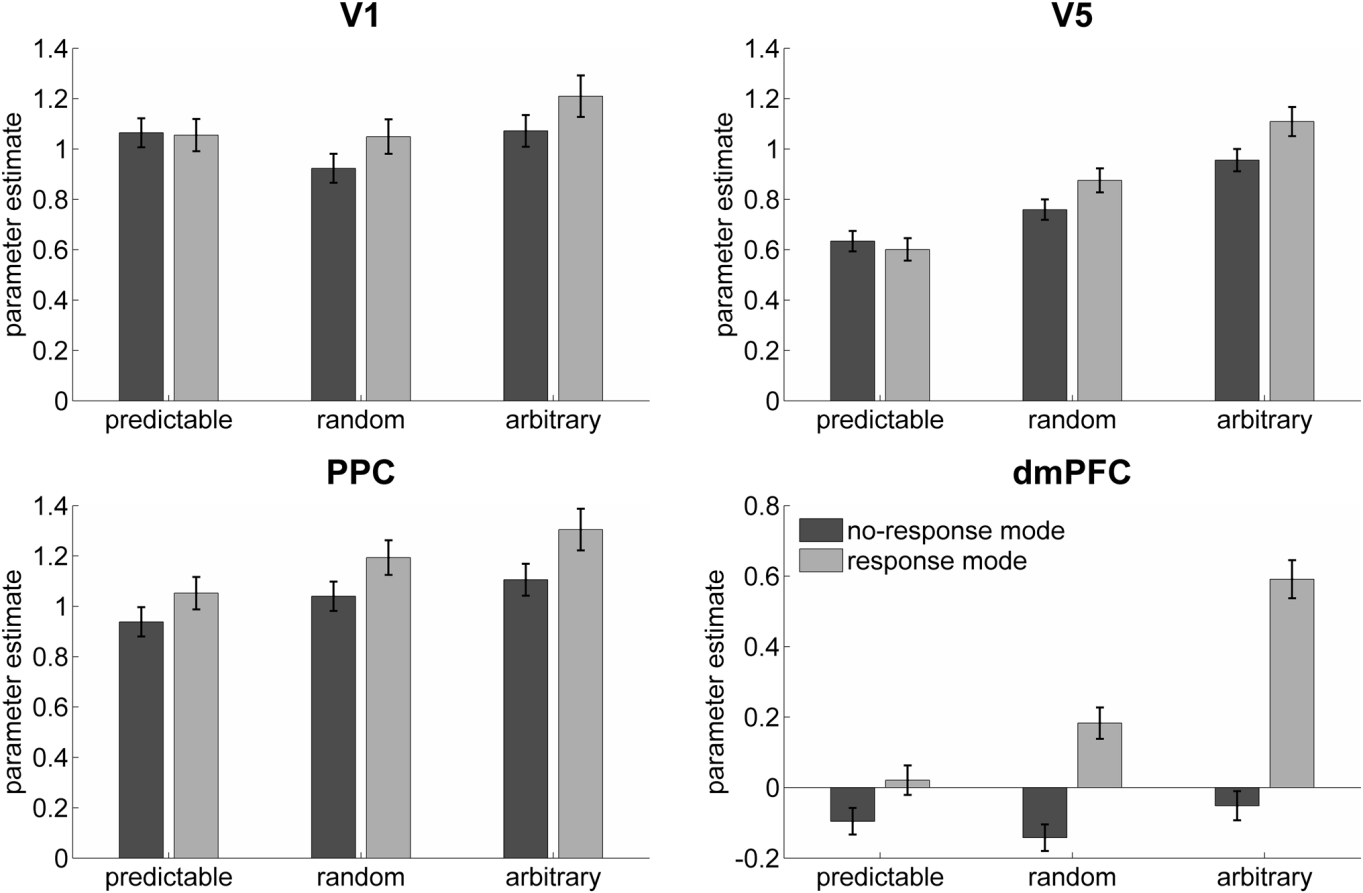
Parameter estimate plots of the three regions of interest (*V1*, *V5* and *PPC*) and the dmPFC. *Bars* indicate the parameters for each condition (implicitly compared to low-level baseline) and *error bars* indicate the *standard error*. The three conditions (predictable, random and arbitrary) are shown separately for the run without motor response (*dark gray*) and for the run with overt motor response (*light gray*). *PPC* posterior parietal cortex; *dmPFC* dorsomedial prefrontal cortex

Moreover, two clusters were located symmetrically at the border between FFG and crus I of the cerebellum, where local maxima were found in both of these structures. The pattern of this interaction was similar across the reported supra-threshold areas with similar activation levels during the predictable and random conditions and slightly more response in the arbitrary block under the no-response session. In the session with overt motor response, however, a quite strong increase in activation was observed with decreasing predictability (see Fig. 5 lower right panel showing the dmPFC as example). Because the interactions reported above were thresholded quite conservatively, we specifically looked for uncorrected effects of response-mode (either as interaction or as main effect) in the three regions of interest for the DCM analyses by extracting the test statistic for the interaction at the local maximum of the motion contrast (V1: *x* = 8, *y* = −90, *z* = 2; V5: *x* = 48, *y* = −66, *z* = 2; PPC: *x* = 20, *y* = −58, *z* = 62). Significant interaction effects were found for V1 (*F*_2,204_ = 3.07, *p* = 0.049) and V5 (*F*_2,204_ = 8.87, *p* = 0.003). For the PPC the interaction was not significant (*F*_2,204_ = 0.78, *p* = 0.461), whereas the main effect of response-mode was (*F*_1,204_ = 6.45, *p* = 0.012). The main effect of response-mode did not reach significance for V1 (*F*_1,204_ = 1.96, *p* = 0.164) nor for V5 (*F*_1,204_ = 3.44, *p* = 0.065). Finally, it should be noted that when rearranging the data, so that the interaction reflects the chronological sequence of the runs rather than the response-mode, then the respective predictability × sequence interaction yielded no suprathreshold voxels even when lowering the *p* threshold to 0.05 corrected with no extent threshold.

### Bayesian model selection among dynamic causal models

**Fig. 6:**
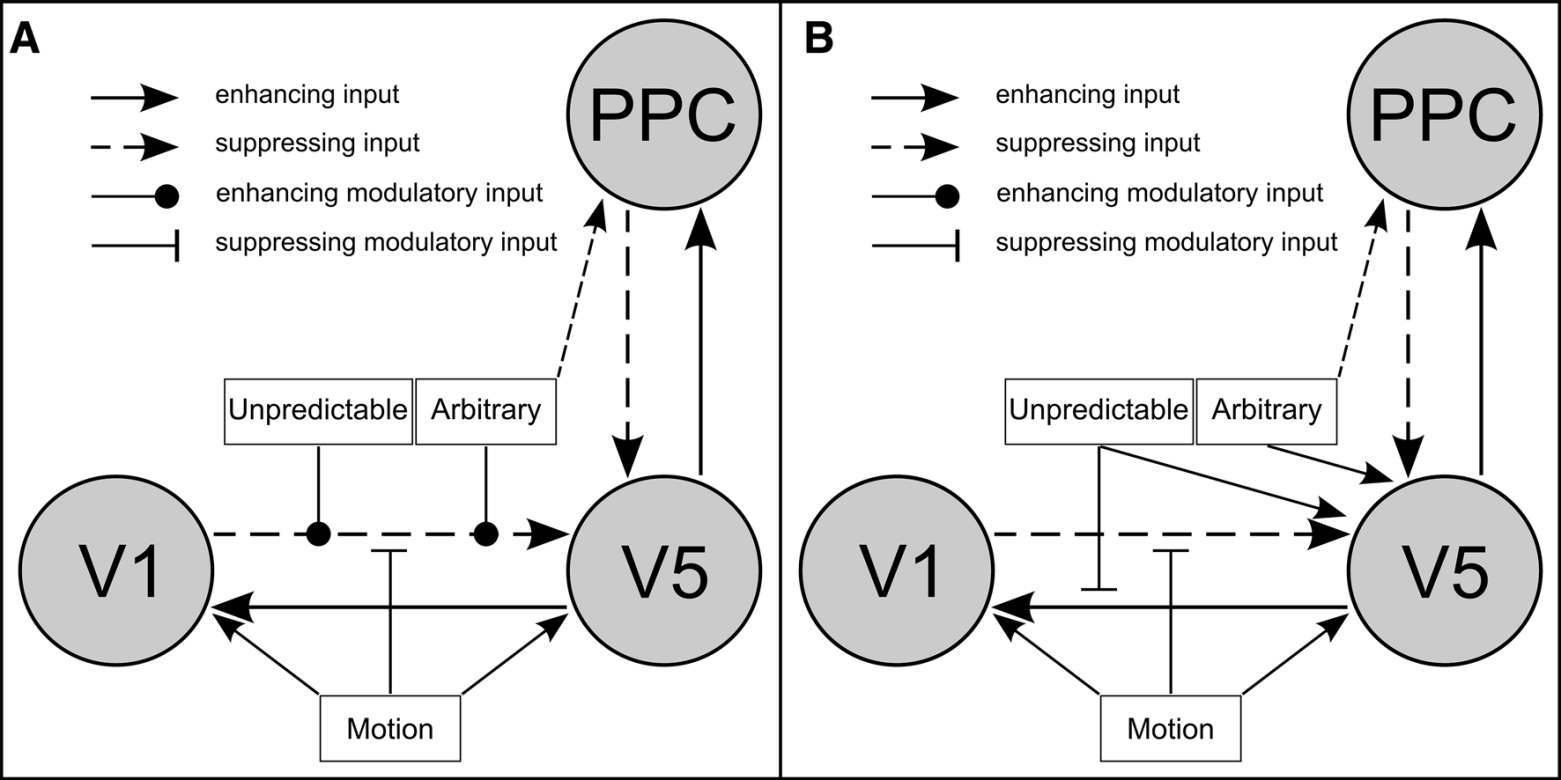
Winning models according to the Bayesian model selection for the two response modes. The *left panel* shows the structure of the winning model for the run with overt motor response and the right panel illustrates the structure of the winning model for the run without motor response. Note that the modulatory input of UNPREDICTABLE on the connection from *V5* to *V1* results from the suboptimal model within Occam’s window and is not significantly different from zero. *PPC* posterior parietal cortex

Since the present study investigates a comparatively simple, perceptual task there is no need to expect, for example, different cognitive strategies between subjects. Hence, for the Bayesian model selection (BMS) procedure we assume that the subjects do not differ with respect to the model structure that caused the data, so that we based the inference method on fixed-effects. Due to the interaction effect on the selected regions—at least to a moderate extent—the two sessions varying the response-mode were not treated as being replications of each other, for which reason the BMS was performed separately for each mode. For the ACTIVE session the BMS resulted in a single model being clearly superior to all other ones as indicated by its posterior probability which was close to 1. The structure of this model was characterized by driving inputs of MOTION on V1 and V5 and a perturbation of PPC by the ABRITRARY condition. As common to all models the V1 → V5 connection was modulated by MOTION, although the negative sign for this parameter was not expected. Moreover, both the UNPREDICTABLE stimuli and the ARBITRARY ones exerted an enhancing modulatory effect on the connection from V1 to V5 (see Fig. 6a).

Posterior Probabilities of the PASSIVE session did not support a single model. Instead two models have been found to be in Occam’s window with posteriors of 80.79 % and 19.15 %, respectively. The model with the higher probability neither had a modulatory input for UNPREDICTABLE nor one for ARBITRARY (the modulatory effect of MOTION on the V1 → V5 connection was common to all models). The other, less likely model showed a slightly suppressing modulatory effect of the UNPREDICTABLE conditions on the backward connection from V5 to V1. Both models had all driving inputs in common with MOTION entering in V1 and V5, UNPREDICTABLE driving V5 only, and ARBITRARY perturbing V5 and PPC (see Fig. 6b). The mean coupling parameters along with their standard errors as calculated by Bayesian parameter averaging are listed in Tables 4 and 5 for the ACTIVE and PASSIVE response-mode, respectively.

**Table 4:** Coupling parameters of the winning model for the active response-mode

To\from	V1	V5	PPC
Average connectivities			
V1	−0.61 (±0.02)	0.62 (±0.04)	–
V5	−0.43 (±0.12)	−0.24 (±0.03)	−0.59 (±0.08)
PPC	–	1.05 (±0.04)	−0.69 (±0.02)

To\from	Motion	Unpredictable	Arbitrary
Driving inputs			
V1	0.09 (±0.02)	–	–
V5	1.02 (±0.05)	–	–
PPC	–	–	−0.10 (±0.01)
Modulatory inputs			
Motion	V1 → V5		−0.51 (±0.09)
Unpredictable	V1 → V5		0.28 (±0.04)
Arbitrary	V1 → V5		0.16 (±0.05)

Numbers indicate the mean of the respective parameter and numbers in brackets refer to their standard deviation

**Table 5:** Coupling parameters of the winning model for the passive response-mode

To\from	V1	V5	PPC
Average connectivities			
V1	−0.60 (±0.02)	0.69 (±0.11)	–
V5	−0.36 (±0.16)	−0.28 (±0.03)	−0.72 (±0.18)
PPC	–	0.95 (±0.06)	−0.65 (±0.03)

To\from	Motion	Unpredictable	Arbitrary
Driving inputs			
V1	0.03 (±0.04)	–	–
V5	1.05 (±0.06)	0.11 (±0.04)	0.15 (±0.03)
PPC	–	–	−0.11 (±0.01)
Modulatory inputs			
Motion	V1 → V5		−0.40 (±0.11)
Unpredictable	V5 → V1		−0.03 (±0.07)

Numbers indicate the mean of the respective parameter and numbers in brackets refer to their standard deviation

A closer inspection of the parameters revealed that the corrected confidence intervals for most parameters did not contain zero. The correction was performed according to the 13 parameters that were tested for each response-mode and was based on the respective quantiles of the sampling from the DCM posteriors as implemented in Bayesian model averaging in SPM. There were three parameters that did not differ significantly from zero in the above mentioned sense which all belonged to the model for the no-response mode: the average coupling parameter from V1 to V5, the direct input of the motion condition on V1 and the modulatory input of the unpredictable condition on the V5 → V1 connection.

## Discussion

The present study was designed to test hypotheses about hierarchical predictive processing in the visual system according to pertinent theoretical assertions. These specific predictions within a small and circumscribed network are discussed in the following section, whereas the results obtained at the level of the whole brain are reconsidered afterwards.

### Hierarchical predictive processing in the visual cortex

According to the model of hierarchical predictive processing in the brain, the information flow from lower hierarchical regions to higher ones should be pronounced with decreasing predictability, because of a larger prediction error for unpredictable stimuli that is passed up the hierarchy (e.g., Clark 2013; Friston 2005). The present study tested this hypothesis using DCM for fMRI in a quite large sample of healthy volunteers performing a predictability of visual motion task. Bayesian model selection indicated quite strong support in favor of the predictive processing hypothesis in that the winning model of the condition requiring motor responses exhibited enhancing modulatory inputs of unpredictable and arbitrary stimulus types on the forward connection from V1 to V5. This effect may reflect increased bottom-up information processing from V1 to V5 during unpredictable visual motion, which is probably due to an enhanced transmission of prediction error. It should be emphasized that the modulatory input of unpredictable stimuli included both random and arbitrary motion. Hence, the modulatory effects of unpredictable (random and arbitrary) as well as arbitrary motion constitute an increase in this forward connection according to the three levels of increasing unpredictability.

The BMS for the passive condition, however, did not corroborate this pattern, although the resulting connectivity structure did not contradict the idea of predictive processing. Instead of an enhancement of the forward connection, we observed a slightly suppressing input of unpredictable stimuli on the backward connection from V5 to V1. Assuming that backward connections originate in “representation units” in deep cortical layers of the hierarchically higher region and terminate mainly in “error units” in superficial layers of lower regions (Mumford 1991), the observed backward suppression might reflect the inability of representation units in V5 to explain away the prediction error that is generated by error units in V1. Nonetheless, it must be noted that the (Bayesian averaged) coupling parameter in question was close to zero because the more parsimonious model without that modulatory input had a far higher posterior (approx. 80 %) as compared to the second model within Occam’s window which comprised this input (approx. 19 %).

Now the question arises why the two response-modes of the same task yielded so different results. One reason for this might be that at least some of the subjects readily digress from the actual task when behavioral performance seems less important for task completion. This implies a diminished compliance of the subjects (be it intentional or not) to stay on track when their effort is not directly observable. In the free-energy formulation of attention this is equivalent to a reduction of precision at the sensory level, which would result in less propagation of prediction error, because of increased uncertainty and hence reduced sensitivity to sensory signals (Feldman and Friston 2010). This is exactly what we observed in the DCM analyses of the no-response run. Another view on this effect, which can be regarded as complementary to the above mentioned argument, assumes additional networks to be involved for the same task when an overt motor response is required.

The fact that the predictability × response-mode interaction yielded a network, whose nodes also manifest a main effect of predictability, corroborates this notion. Although the subjects in the present study only reacted in response to—as opposed to act on—the stimuli, the differential processing of the same stimuli in the brain may be related to a resonating effect of (any kind of) motor output that presumably underlies active inference (Friston 2010; Limanowski and Blankenburg 2013). This interpretation implies that, regardless of the ability to manipulate the external world, any motor response has a non-negligible impact on the processing of external stimuli. Most likely, a more comprehensive picture of the observed differences requires an extensive modeling of other important nodes on the one hand, e.g., the dorsomedial prefrontal cortex (Regenbogen et al. 2013), the thalamus (Saalmann and Kastner 2011), or the cerebellum (Kellermann et al. 2012). On the other hand, refinements of experimental manipulation of response-modes need to be devised to differentiate potential effects that any diverse motor outputs might have (e.g., Warbrick et al. 2013). In conclusion, the BMS results of the no-response run should be considered with caution because the interim winning models do not seem to be quite plausible. This finding either suggests that our model space did not include a useful model for this session or that fixed-effects BMS might be untenable for this task because lacking behavioral relevance leads to the above mentioned decline in the subjects’ compliance.

The concepts of behavioral relevance (as differentially induced by the response-modes) and predictability may share a key effect that both exert on the nervous system, namely attention. The idea that attention should be rather regarded as an effect rather than a cause has been elaborated by Anderson (2011). Accordingly, (goal-directed) attention can be regarded as a consequence of behavioral relevance which is implemented in a top-down fashion, whereas unpredictability gives rise to (stimulus-driven) attention due to the salience of the stimulus in a bottom-up manner. In this sense stimulus-driven attention seems to be confounded in the present study, because more attentional resources are presumably allocated to processing unpredictable stimuli. Although the decision between cause or effect of attention cannot yet be made, the notion of attention as being rather an effect seems reasonable when unpredicted or salient stimuli are presented. Therefore, we argue that a decline in predictability inevitably goes along with more salience and stimulus-driven attention. An amalgamated representation of priority was proposed by Fecteau and Munoz (2006) to combine bottom-up effects induced by salience and top-down effects that determine the relevance of stimuli. The authors conclude that the combined representation of an object’s distinctiveness and its relevance to observers in so called priority maps is likely instantiated in the oculomotor system (Fecteau and Munoz 2006), underscoring the need for an extension of the relevant network.

Nevertheless, Kok et al. (2012) recently demonstrated that goal-directed attention can be manipulated orthogonally to predictability. Beyond that, the study has shown that directed spatial attention can reverse the attenuating effects of predictability on sensory processing (Kok et al. 2012). The present study, however, was designed to investigate the effects of predictability of perceptual properties with goal-directed attention held constant (although the response-mode may have implicitly changed goal-directed attention via behavioral relevance or priority). In its free-energy formulation attention is considered to be the process of optimizing the synaptic gain to represent sensory precision (Feldman and Friston 2010). Although this phrasing rather emphasizes a top-down control of attention the net effect with respect to hierarchical predictive processing is the same in relation to processing unpredicted or salient stimuli. Whereas goal-directed attention increases the synaptic gain of representation units to inputs from error units, salience directly increases the input from lower to higher regions, both leading to an amplification of prediction errors. This distinction between these two complementary processes may be the reason for the fact that—contrary to Kok et al. (2012)—we did not find evidence for a modulation of the self-connection of V5 for unpredictable stimuli. Moreover, it is important to note that a modulation of this self-connection is ambiguous with respect to hierarchical processing because an increase of the synaptic gain of V5 in our models can be associated with enhanced responsiveness to both forward inputs from V1 as well as backward projections from PPC.

Apart from explaining perceptual and cognitive phenomena on a neuronal level, one of the central claims of the theory of hierarchical predictive processing is its ability to provide neuronal mechanisms able to describe phenomena observed in pathological and particular psychiatric circumstances. For instance, an aberrant prediction error has been associated with schizophrenia (Adams et al. 2013). According to this view, a reduction in the precision of prior beliefs (or top-down predictions), relative to sensory evidence (or bottom-up prediction error) may lead to abnormalities observed in schizophrenia, e.g., psychotic symptoms, cognitive deficits or negative symptomatology. Another psychiatric disease which has been tried to understand in terms of hierarchical processing is autism spectrum disorder (ASD). Two former theories—namely weak central coherence (WCC; Happé and Frith 2006) and enhanced perceptual functioning (EPF; Mottron et al. 2006)—separately emphasized reduced global processing (in case of WCC) or enhanced local processing (in case of EPF) observed in ASD. A predictive coding perspective may unify these accounts in the sense that an overemphasis of the prediction error or overly high precision expectation in sensory input may explain both of these observed effects (Van de Cruys et al. 2014; Lawson et al. 2014; Palmer et al. 2015a, b).

We envisage an application of the task presented in this study to patients with schizophrenia and ASD. Although both disease patterns are associated with an enhanced forward passing of prediction errors, there are differential hypotheses according to the predictive coding perspective. Because in schizophrenia prior beliefs are assumed to be reduced, one would expect enhanced forward coupling of different sensory levels (V1 and V5) for all conditions with a diminished differentiation according to predictability. Contrariwise, ASD is rather associated with an excessively high precision expectation of sensory input which hypothesizes an augmented differential response in the coupling from lower to higher visual regions as a function of predictability of visual motion.

### Whole brain GLM analyses

The results for the main effect predictability exhibited a large distributed network that bore at least some resemblance to the goal-directed and stimulus-driven attention network, which is associated with dorsal and ventral fronto-parietal areas, respectively (Corbetta and Shulman 2002). While our data lend only partial support for the goal-directed attention stream with dorsal engagement in the parietal lobe (e.g. in PPC) and in the FEF, the activation pattern of the main effect of predictability provides quite strong evidence in favor of the rather right-lateralized involvement of the ventral fronto-parietal network assumed to underlie stimulus-driven attention. The right inferior frontal gyrus has repeatedly been linked to novelty detection (e.g., Dobbins and Wagner 2005; Gur et al. 2007) and might play an important role—together with the (TPJ)—as a circuit-breaker during reorienting to spatially unexpected targets (Corbetta and Shulman 2002).

The conjunction of the two contrasts indicates that both regions, right inferior frontal gyrus and right TPJ, are more or less parametrically linked to (un-) predictability in the present study. The contrast ARBITRARY > RANDOM revealed additional cortical activation in the dmPFC as well as subcortical clusters in the thalamus and brainstem. Although the limited spatial resolution of fMRI scans prohibits a definite assignment of activations to distinct subcortical nuclei, the peak activity in the brainstem may be attributed to the superior colliculi, whereas the thalamic engagement may originate from the pulvinar (Petersen et al. 1987), the reticular nucleus (Sturm et al. 1999; Kellermann et al. 2011), and/or the intralaminar nuclei (Yeo et al. 2013). The superior colliculus is part of the oculomotor network and has been—like the pulvinar—associated with salience (Robinson and Petersen 1992; Fecteau and Munoz 2006). However, the role of the superior colliculus has been ramified because of its relation to inhibition of return. Inputs of bottom-up salience and top-down relevance seem to converge in the superior colliculus (albeit during different stages of processing) for which reason Fectau and Munoz (2006) proposed the term priority-map to merge the two. A recent study suggests that the superior colliculi are indeed influenced by top-down signals from lateral prefrontal cortex (Everling and Johnston 2013).

Even though we anticipated differences in activations between the two response-modes in motor related areas (not reported), we did not expect to find noteworthy effects of the response-mode on different levels of the predictability factor. Yet such differences between response-modes have been reported in a recent study, where subjects also performed a session in which they counted the number of targets in addition to a passive and a response condition (Warbrick et al. 2013). In general, stronger activation of the dmPFC (extending to SMA) is associated with tasks requiring overt motor as opposed to non-motor responses (Langner and Eickhoff 2013). This structure has been proposed to serve as brake to maintain a preparatory motor-set which is inhibited at the same time so as to avoid premature responses. Gradually releasing this break would trigger the prepared response when a certain threshold is exceeded (Eichele et al. 2008; Danielmeier et al. 2011; Langner and Eickhoff 2013). Based on this we assume that the arbitrary condition activates a preparatory motor-set which is inhibited by the dmPFC.

Because of the (relative) anticipatory certainty of upcoming targets (i.e., movement changes) in the predictable but also in the random condition, the response-set is not pre-activated to the same extent as compared to the arbitrary blocks where targets occur any time. The temporal control of motor responses might be arranged more efficiently in predictable blocks without simultaneous motor preparation and inhibition. Crus I of the cerebellum may provide timing information of perceptual events (O’Reilly et al. 2008; Kellermann et al. 2012), which might be enhanced during the session requiring motor responses. The involvement of the inferior frontal gyrus (close to the inferior frontal junction) in the interaction is in line with its presumed role in setting up stimulus–response mappings (Hartstra et al. 2011; Langner and Eickhoff 2013). The thalamus is a key structure in the ascending reticular activating system (Yeo et al. 2013) as relay from the reticular formation to the cortex so as to generate and maintain an adequate arousal level (Hasselmo and Sarter 2011). It is conceivable that unpredictable and therefore, salient stimuli in the arbitrary blocks generate a high arousal which is even facilitated by response requirements.

In summary, the present study is not designed to separate different stages of processing by a mere cognitive subtraction strategy. Nevertheless, we hope to have shown that the task yields robust activations in almost all well-known areas assumed to support (visual) attention, including the dorsal and ventral parietal network as well as subcortical structures like the thalamus and superior colliculi. Importantly, an overt motor response seems to have an amplifying and/or modifying effect on processing in other regions, even if these are indirectly or not at all related to motor output. Therefore, this task seems to be well suited to characterize the functional integration of circumscribed attentional networks with dynamic causal modeling. Unfortunately, this characterization is beyond the scope of the present study, because we aimed at testing specific hypotheses regarding hierarchical predictive processing considered above.

### Limitations

Besides aforementioned constraints regarding, for example, overt motor output there are other limitations of the present study that merit consideration for future work. The most severe constriction of the stimuli at hand is the number of changes in motion direction, which differs substantially between the arbitrary condition [with a mean (*M*) of 28.9 changes per block and standard deviation (STD) of 3.9] and the other two experimental manipulations [predictable (*M* = 18.5, STD = 1.0) and random (*M* = 18.3, STD = 2.6)]. There are three possibilities to overcome this limitation, although each one has other drawbacks which we judged more severe in relation to the compromise we made: Two possibilities comprise either a reduction of the duration of the stimuli or a deceleration of motion during the arbitrary motion condition to adapt the number of direction changes. A third option would be downsizing the frame in the other two conditions such that predictable and random changes in motion direction occur more often. These differences in motion direction changes are accompanied by differences in motor reaction regarding button presses (when a reaction was required) as well as saccadic eye movements. The latter will likely be associated with activations of the superior colliculi and frontal eye fields, where particularly the latter have an influence on the visual cortex (Heinen et al. 2014). As described in the methods (“Dynamic causal modeling”) we assumed that putative top-down effects from other regions (e.g., those mentioned above) may be captured as direct inputs of unpredictable or arbitrary stimuli on either V5 or PPC. The strong evidence which we found during the pre-selection for a direct input of the arbitrary condition on PPC may reflect an effect that is mediated by structures like the frontal eye fields. However, we did not find evidence for a direct effect of the arbitrary condition on V1 or V5, indicating that the above mentioned effects are mediated by the PPC at least in the present study. Nevertheless, this interpretation remains speculative unless the respective candidate regions like the frontal eye fields or superior colliculi are not included in the models under consideration. Moreover, the confounding effects of eye movements and number of motion direction changes in the arbitrary condition remain a limiting concern of the present study which should be addressed in future studies by changes in the stimuli as mentioned above.

Although we already broached the issue of the limited number of nodes included in the DCM analysis, it must be pointed out that any change in the system may result in systemic effects on the whole network, i.e., coupling parameters depend on the structure of the whole model. We surmise that such an effect may have occurred to the endogenous connectivity from V1 to V5, which turned out to be negative for the winning models. If our assumption is correct, the direct input of motion to V5 (via the lateral geniculate body) is possibly overestimated because we did not constrain this input with any prior weights. Such a weighting, however, may yield physiologically more plausible results since the proportion of cells projecting from the lateral geniculate nucleus to V5 is only about 10 % of those compared to the population in V1 that innervates V5 (Sincich et al. 2004).

Whereas questions regarding the absence or presence of connections between nodes or the impact of direct or modulatory inputs can be addressed by extending the model space for a Bayesian model selection accordingly, the question of including a node or not cannot be addressed by model selection (at least for fMRI data). The reason is that a comparison of different models requires the same data to be subjected to each model and inclusion or exclusion of a region is equivalent to adding or removing the data of that node, respectively.

## Conclusions and outlook

The present study was designed to test specific hypotheses about enhanced feed-forward connectivity in the visual cortex in response to unpredictable visual motion. These predictions rest upon the notion of hierarchical predictive processing, which forms the basis of the Bayesian brain hypothesis (e.g., Clark 2013; Friston 2010). Importantly, the patterns of effective connectivity strongly supported these predictions when the stimuli were behaviorally relevant. Hence, the quite simple visual task presented in this study seems to be well suited to further investigate hierarchical predictive processing in and beyond the visual cortex so as to include other regions related to motor planning and execution as well. Moreover, the present task may be indicative of trait abnormalities in patients suffering from psychiatric disorders or yet even in their relatives. A recent review suggested that psychotic symptoms may be the result of an imbalance (in the precision) of feed-forward and backward connections between hierarchical levels of processing, presumably underlying known effects like an attenuated mismatch negativity, impaired smooth pursuit eye movements or a weaker force-matching illusion (Adams et al. 2013). In conclusion, the present study lends empirical support for hierarchical predictive processing in accord with the predictability of visual motion, for which reason the present task seems to be well suited to shed light on putatively disturbed effective connectivity in psychiatric disorders.
